# Structural basis for cpSRP43 chromodomain selectivity and dynamics in Alb3 insertase interaction

**DOI:** 10.1038/ncomms9875

**Published:** 2015-11-16

**Authors:** Annemarie Horn, Janosch Hennig, Yasar L. Ahmed, Gunter Stier, Klemens Wild, Michael Sattler, Irmgard Sinning

**Affiliations:** 1 Heidelberg University Biochemistry Center (BZH), INF 328, Heidelberg D-69120, Germany; 2 Center for Integrated Protein Science Munich at Biomolecular NMR Spectroscopy, Department Chemie, Technische Universität München, Lichtenbergstrasse 4, Garching DE-85747, Germany; 3 Institute of Structural Biology, Helmholtz Center Munich, Ingolstädter Landstrasse 1, Neuherberg D-85764, Germany

## Abstract

Canonical membrane protein biogenesis requires co-translational delivery of ribosome-associated proteins to the Sec translocase and depends on the signal recognition particle (SRP) and its receptor (SR). In contrast, high-throughput delivery of abundant light-harvesting chlorophyll *a,b*-binding proteins (LHCPs) in chloroplasts to the Alb3 insertase occurs post-translationally via a soluble transit complex including the cpSRP43/cpSRP54 heterodimer (cpSRP). Here we describe the molecular mechanisms of tethering cpSRP to the Alb3 insertase by specific interaction of cpSRP43 chromodomain 3 with a linear motif in the Alb3 C-terminal tail. Combining NMR spectroscopy, X-ray crystallography and biochemical analyses, we dissect the structural basis for selectivity of chromodomains 2 and 3 for their respective ligands cpSRP54 and Alb3, respectively. Negative cooperativity in ligand binding can be explained by dynamics in the chromodomain interface. Our study provides a model for membrane recruitment of the transit complex and may serve as a prototype for a functional gain by the tandem arrangement of chromodomains.

## Abstract (web summary)

The chloroplast signal recognition particle delivers LHCPs to the thylakoid membrane by interaction of cpSRP43 with the Alb3 insertase. Here the authors decipher the specific recognition of the Alb3 C-terminal tail within the interface of two communicating chromodomains by structural biochemistry.

## Graphical abstract

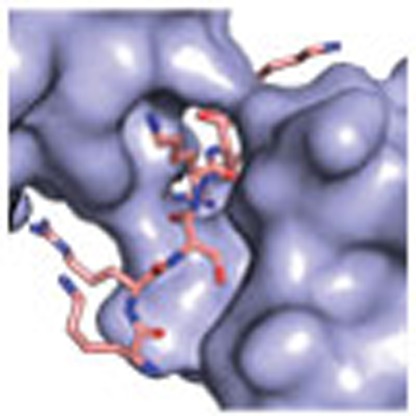

Co-translational membrane protein delivery relies on the signal recognition particle (SRP) machinery found in the cytosol of all prokaryotes and eukaryotes^1^,^2^,^3^. The SRP core is universally conserved and consists of SRP54 (Ffh in bacteria) and the SRP RNA. In contrast to cytosolic SRPs, SRP in the chloroplasts of higher plants (cpSRP) has lost its RNA for targeting proteins to the thylakoid membrane^4^,^5^ and acts both in a co- and post-translational manner. Co-translational targeting of chloroplast-encoded cargo proteins is carried out by cpSRP54 alone, whereas the post-translational cpSRP transport pathway is mediated by cpSRP54 in complex with cpSRP43, a 43-kDa protein that is unique to chloroplasts^6^,^7^,^8^,^9^.

Post-translational cpSRP-dependent protein transport is a dedicated pathway for members of the light-harvesting chlorophyll *a,b*-binding protein family (LHCPs)^10^, the most highly abundant membrane protein in chloroplasts. LHCPs serve as antenna systems in photosynthesis^11^ to funnel absorbed light energy to the photoreaction centres. They contain three transmembrane helices (TM1 to TM3) to which chlorophylls and carotenoids bind during insertion into the thylakoid membrane^12^. Synthesized on cytosolic ribosomes, the nuclear-encoded LHCPs are guided to the chloroplast envelope by a cleavable transit peptide and are imported into the chloroplast stroma via interaction with the TOC/TIC import machinery^13^,^14^. In the stroma, the LHCPs are transferred from the envelope via the small LTD protein^15^ to a soluble transit complex with cpSRP^7^. The transit complex is guided to the thylakoid membrane, where the LHCPs are inserted by the interaction with the membrane-bound SRP receptor cpFtsY and the C terminus of the membrane insertase Alb3 (refs 16, 17) (Fig. 1a). Alb3 belongs to the YidC/Oxa1/Alb3 family of membrane insertases^18^,^19^, which are responsible for insertion and folding of membrane proteins and their assembly into larger protein complexes^18^. *Escherichia coli* YidC represents the best characterized member of this family with its C terminus being involved in ribosome interaction^20^,^21^,^22^.

The C terminus of Alb3 is intrinsically disordered, recruits cpSRP43 to the thylakoid membrane, and participates in cpSRP-dependent post-translational membrane targeting^23^. CpSRP43 is a modular protein with a unique arrangement of three chromodomains (CD1–3) and four ankyrin repeats (Ank1–4)^24^,^25^ (Fig. 1a,b). Chromodomains and ankyrin repeats are versatile protein–protein interaction modules and allow cpSRP43 to participate in numerous, specific interactions with linear motifs. Chromodomains are typically found in the nucleus where they play a key role in chromatin remodelling and gene expression^26^,^27^ like the heterochromatin protein 1 and polycomb^28^. The chromodomains of these proteins accommodate methylated lysines within an ARKS signature sequence of histone tails in an ‘aromatic cage'. In contrast, cpSRP43 CD2 binds a positively charged arginine-rich motif at the C-terminal tail of cpSRP54 in a twinned and modified cage and thereby recruits cpSRP54 into the transit complex^25^. Ankyrin repeats are typical tandem-arrays of 33 residues that bind linear motifs in different biological contexts and are also used for the design of specific binding proteins (DARPins)^29^. In cpSRP43, the ankyrin repeats bind a conserved region in the loop between LHCP TM2 and TM3 (the L18 motif)^24^,^30^. This enables cpSRP43 to act as a specific chaperone that prevents LHCP aggregation^31^,^32^. Recent studies showed cpSRP43 to exhibit significant inter-domain dynamics, which is reduced on cpSRP54 binding^33^.

We previously showed that the two C-terminal chromodomains CD2 and CD3 of cpSRP43 are important for binding two positively charged motifs in the Alb3 C-terminal tail (A3CT)^23^. However, the molecular details of the cpSRP43–A3CT interaction and the selectivity, dynamics and cooperativity with respect to cpSRP54 binding remained unknown. Here we present a detailed structural and biochemical analysis of the cpSRP43 CD2CD3 interaction with A3CT, demonstrating that CD3 binds to a linear motif of A3CT and that LHCP targeting is regulated by a serial connection of ankyrin repeats and chromodomains. Our data provide the structural basis for transit complex tethering to the thylakoid membrane by the Alb3 insertase.

## Results

### Alb3 binds specifically to cpSRP43 CD3

**Figure 1:**
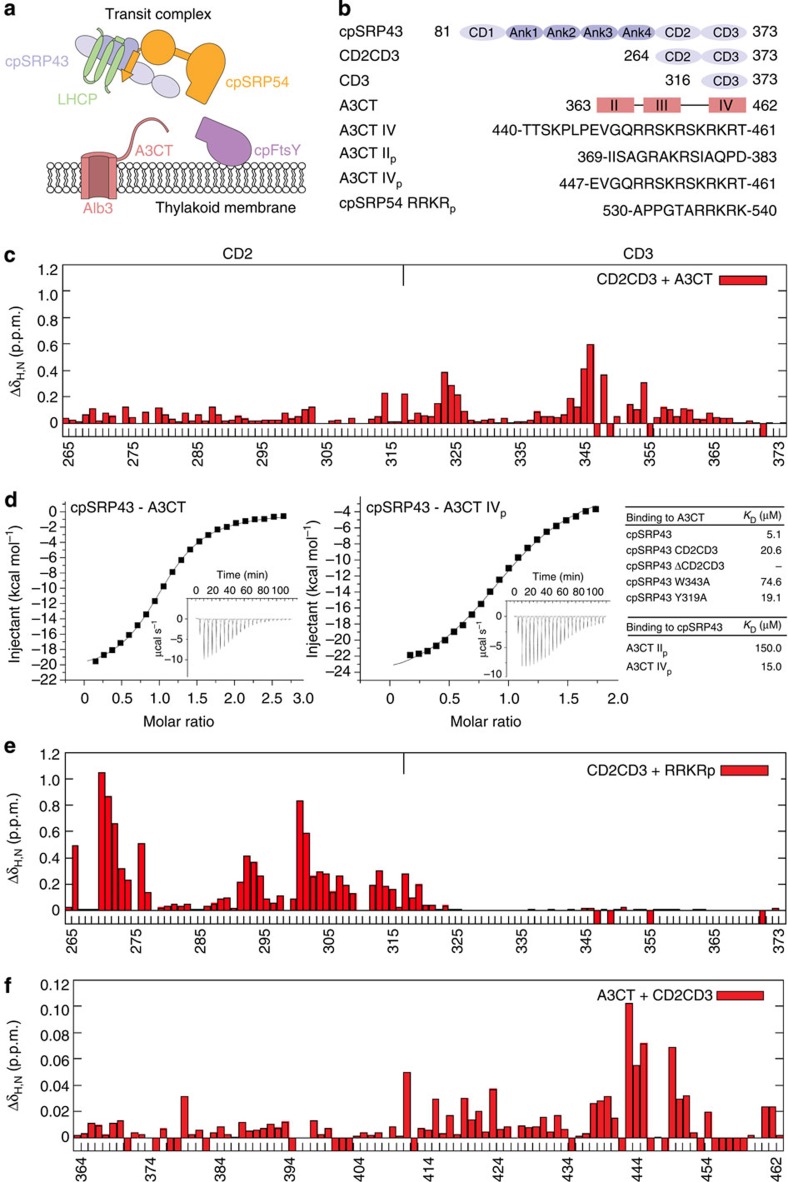
NMR and ITC analysis of the cpSRP43–A3CT interaction. (**a**) Scheme of the cpSRP system in post-translational LHCP targeting. (**b**) Schematic representation of cpSRP43 and A3CT constructs used in this study. (**c**) NMR chemical shift perturbations (CSPs) of CD2CD3 on titration with A3CT. Major CSPs are observed only for residues located in CD3. (**d**) Characterization of the interaction between cpSRP43 and A3CT. ITC titrations are shown for cpSRP43 with A3CT (left) and cpSRP43 with A3CT IV_p_ (middle). *K*_D_ values for relevant ITC measurements are listed in a table (right). (**e**) CSPs of CD2CD3 on titration with RRKR_p_. Major spectral changes on RRKR_p_ binding occur only for residues located in CD2. (**f**) CSPs observed in A3CT on titration with unlabelled CD2CD3. Significant CSPs are observed for residues located at the C terminus of A3CT (motif IV and flanking residues). CSPs with a negative value indicate residues that could not be assigned in the bound state.

Recruitment of the transit complex to the thylakoid membrane involves the conserved interaction of the A3CT with the two C-terminal chromodomains of cpSRP43 (in the following denoted with CD2 and CD3 only)^23^ (Supplementary Fig. 1a,b). To test whether A3CT is able to discriminate between CD2 and CD3, we used nuclear magnetic resonance (NMR) spectroscopy to characterize the interaction with a cpSRP43 construct comprising CD2 and CD3 (CD2CD3) (Fig. 1b). Titration of unlabelled A3CT into ^15^N and ^13^C labelled CD2CD3 shows NMR chemical shift perturbations (CSPs) for residues located almost exclusively in CD3, while CD2 was only affected at its C-terminal end (residues 313 and 316) (Fig. 1c). These data show that A3CT binds to cpSRP43 CD3 and does not directly compete with cpSRP54 for CD2. To quantify the binding event, we performed isothermal titration calorimetry (ITC, Supplementary Table 1a). CD2CD3 binds A3CT with a dissociation constant of 20.6 μM in contrast to 5.1 μM observed for full-length cpSRP43 (Fig. 1d), indicating a stabilizing effect of the ankyrin repeats on the chromodomains.

In a crystal structure of a cpSRP43–cpSRP54 complex, we previously reported how CD2 binds the C-terminal tail of cpSRP54 harbouring a RRKR motif^25^. In a titration of CD2CD3 with the same RRKR peptide (RRKR_p_) (Fig. 1b) monitored by NMR, we find that this ligand binds exclusively to CD2 also in presence of CD3, although some CSPs are observed for N-terminal residues in CD3 (Gly316 to Glu318) (Fig. 1e). NMR CSPs for RRKR_p_ binding to CD2 are much stronger than in the NMR titration for A3CT binding to CD3, consistent with the ∼10 times higher binding affinity of the cpSRP54 tail in comparison with A3CT as determined by ITC^25^. The data also explain our previous observations that cpSRP43 is able to bind both ligands at the same time^23^ and show that CD2 and CD3 provide specific interaction sites for the tails of cpSRP54 and Alb3 despite the high sequence similarity between the two chromodomains and the ligands^25^.

### CD3 binds the A3CT motif IV

Having established that A3CT interacts specifically with CD3, we tested whether CD3 discriminates between similar interaction motifs (II and IV) within A3CT. The A3CT harbours three ARKS-like signature sequences that could potentially bind to chromodomains (Supplementary Fig. 1b). From the cpSRP43ΔCD3–RRKR_p_ complex it was known that CD2 accommodates the second arginine in the ARRK signature sequence of cpSRP54 in a modified cage^25^. However, the signature is extended to 531-PPGTARRKR and all three arginines are read-out by cpSRP43. Motif II of A3CT contains a 375-AKRS sequence flanked by predominantly small and uncharged residues, whereas motif IV is highly positively charged and contains two putative overlapping binding sequences (453-SKRS and 456-SKRK). Therefore, it was *a priori* not clear, which of the three signature sequences would bind, whether binding is specific or if there is any promiscuity.

Titration of unlabelled CD2CD3 into ^15^N,^13^C labelled A3CT showed CSPs most pronounced for the C-terminal region of A3CT harbouring motif IV (Fig. 1f). The CSPs are considerably smaller than for the previous titrations, reflecting the lower affinity of A3CT^23^. Based on these data, we designed an A3CT variant comprising only the C-terminal 28 residues including motif IV (A3CT IV peptide, residues 434–461; Supplementary Fig. 2a). Titration of CD3 only into A3CT IV peptide confirmed the specific interaction (Supplementary Fig. 2a). The region including motif II (residues 369–379) did not show any significant CSPs. Titrations of a motif II peptide into double-labelled CD2CD3 or CD3 alone confirmed these results (Supplementary Fig. 2b,c). ITC experiments revealed that A3CT motif II and IV bind to CD2CD3 with dissociation constants *K*_D_ of 150 μM and 15 μM, respectively (Fig. 1d), while the affinity to the complete A3CT corresponds to a *K*_D_ of 5 μM. Thus the cpSRP43–A3CT interaction is in a similar range as canonical chromodomain interactions with histone tails, which typically exhibit *K*_D_ values of 1–10 μM (ref. 34).

Taken together, our NMR and ITC experiments show that the cpSRP43–Alb3 complex is based on the specific interaction of CD3 with the C-terminal region of A3CT harbouring motif IV, and neither CD2 nor A3CT motif II significantly contribute to the interaction.

### Crystal structure of the CD2CD3-motif IV complex

**Figure 2:**
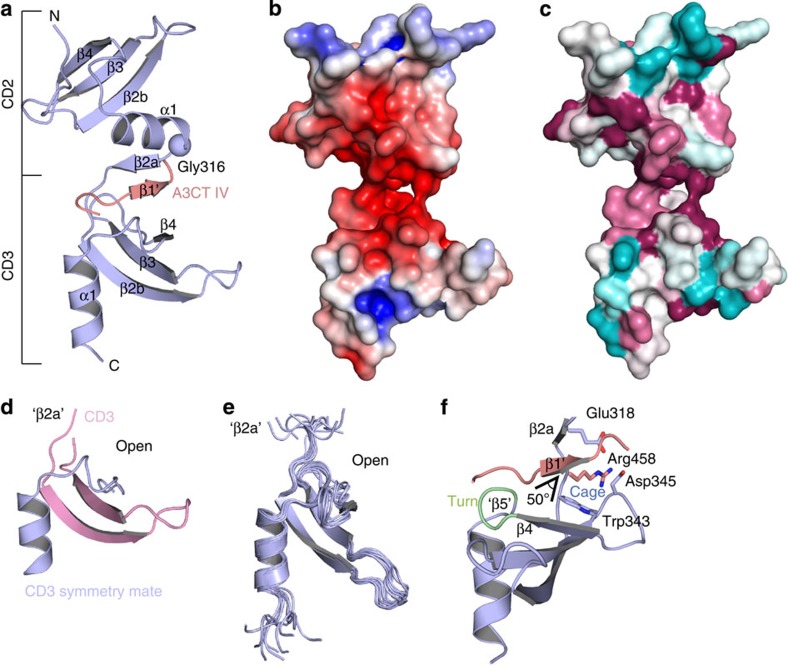
Structural characterization of the cpSRP43–A3CT complex. (**a**) Crystal structure of cpSRP43 CD2CD3 (blue) with bound A3CT IV (salmon) as part of the fusion construct with thioredoxin (not shown). (**b**) The electrostatic surface potential of CD2CD3 is shown with positive charges in blue and negative charges in red (contoured at±5 kT). (**c**) ConSURF analysis showing the degree of conservation mapped on the surface of the CD2CD3 structure. Highly conserved residues are represented in dark red, partially conserved residues in magenta and less conserved residues in cyan. (**d**) Crystal structure of cpSRP43 CD3 in the open conformation with an unstructured ‘β2a' strand. CD3 is stabilized by helix swapping between two CD3 molecules. (**e**) Solution structure of cpSRP43 CD3. A superposition of the 10 lowest energy structures is shown. (**f**) Close-up view of CD3 bound to A3CT IV highlighting the gap between strands β4 and β1′ induced by a helical turn (green) and forming the cage for substrate recognition. The increased β-sheet twist in the β-barrel, resulting in the loss of strand ‘β5' is indicated (50°).

Having identified the specific interaction between CD3 and A3CT motif IV, we set out to characterize the binding mode by NMR and X-ray structure determination. As the interaction of CD3 with A3CT motif IV is rather weak and attempts to crystallize this minimal complex were not successful, we designed a fusion construct with the C terminus of CD2CD3 (residues 264–373) covalently linked (with a triple GS-linker (GS)_3_) to A3CT motif IV (A3CT IV, residues 440–461) (Fig. 1b and Supplementary Fig. 3a), to increase the local concentration and to stabilize the interaction. However, as this construct did not result in crystals and motivated by recent success using carrier proteins in crystallization^35^,^36^, we fused thioredoxin (Trx) with a single GS-linker to the N terminus of CD2CD3 (Supplementary Fig. 3a). This Trx–CD2CD3–IV construct could be crystallized and the structure was solved by molecular replacement at 2.8-Å resolution (Fig. 2a, Table 1 and Supplementary Fig. 3b). The Trx–CD2CD3–IV structure contains two molecules in the asymmetric unit (Supplementary Fig. 3c) and comprises Trx residues 12–119, CD2CD3 residues 265–369 and A3CT residues 453–461. The N terminus of Trx (residues 1–11), the (GS)_3_-linker between CD2CD3 and A3CT IV and residues 440–452 of A3CT IV are disordered. The Trx-carrier is involved in crystal contacts (Supplementary Fig. 3d) as observed for other fusion constructs^36^.

CD2 and CD3 show the typical chromodomain fold consisting of a three-stranded antiparallel β-sheet (strands β2b, β3 and β4) and a perpendicularly packed C-terminal helix α1 (Fig. 2a). The two chromodomains are rotated relative to each other by an angle of about 90° and are directly connected without a linker at Gly316. This tandem chromodomain arrangement exhibits a negatively charged and conserved surface in the CD2–CD3 interface (Fig. 2b,c) that accommodates the highly positively charged motif IV (interface area of about 800 Å^2^). The electronegative CD2CD3 surface extends the previously described charged surface of cpSRP43ΔCD3 that mimicks the SRP RNA, which is absent in cpSRP of higher plants^24^.

CD2 is found in an open (unliganded) chromodomain conformation, whereas CD3 adopts a closed conformation as seen for CD2 in complex with RRKRp^25^ (r.m.s.d. for closed conformations of 1.25 Å for 43 residues, Supplementary Fig. 4d). Closure corresponds to the ordering of the chromodomain N terminus that folds into strand β2a and integrates the ligand as strand β1′ by β-completion. To characterize changes in the conformation and dynamics of CD3 on ligand binding, we also determined the structure of the unliganded CD3 domain by X-ray crystallography and NMR (Fig. 2d,e). Both structures are in the open conformation characterized by the unstructured first strand β2a due to the absence of its binding partner. In the crystal structure, the CD3 fold is stabilized by helix swapping between crystallographic neighbours emphasizing the high flexibility of isolated chromodomains (Supplementary Fig. 3e). Helix swapping is probably due to crystal packing forces as the NMR structure exhibits a compact monomeric chromodomain fold in solution (Fig. 2e and Supplementary Table 2).

**Figure 3:**
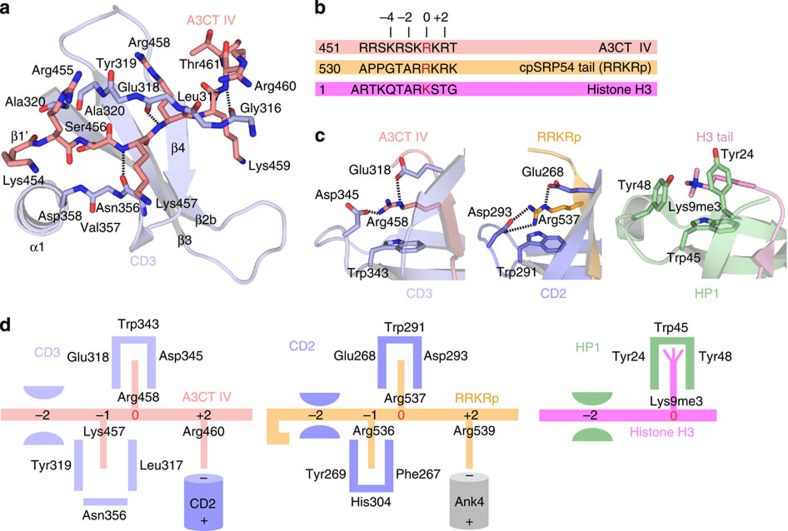
Ligand recognition by chromodomains. (**a**) Close-up view of the backbone interactions of CD3 with A3CT IV. Hydrogen bonds are indicated by dashed lines. (**b**) Sequence alignment of chromodomain ligands. The 0 position binding to the cage is depicted in red. (**c**) Specific read-out of the 0 position in different chromodomains. Left: A3CT IV in CD3, middle: RRKRp in CD2, right: H3K9me3 in HP1. (**d**) Schematic representations of the respective chromodomain–ligand interactions.

The accommodation of the ligand as strand β1′ in CD3 results in the formation of a β-barrel as described for the CD2 interaction with RRKR_p_^25^ (Supplementary Fig. 4b,d). Strand β1′ is hereby sandwiched between strand β2a and a short strand β5 formed by the loop between strand β4 and helix α1. The combined chromodomain–ligand β–barrel is peculiar in the sense that the parallel strands β4 and β5 (the latter not formed in CD3) span only half of the barrel and are laterally offset by an inserted helical turn, thus creating a void in the barrel between strands β4 and β1′, which is filled by the ligand bound to its ‘cage' (Fig. 2f). In the classical chromodomain interaction with histone tails, the cage is formed by three aromatic residues that accommodate the methylated lysine of the tail (Fig. 3c, Supplementary Fig. 4c). In the CD2CD3–IV complex ligand binding is modified for the specific recognition of an arginine residue (see below) and the absence of strand β5 is due to a twist (usually 25° in β-sheets) of strand β1′ with respect to the neighbouring strands β2a (50°) and the ‘β5' region (0°) (Fig. 2f). Therefore, strands β1′ and β2a are shorter as observed in the cpSRP43ΔCD3–RRKR_p_ complex^25^ (Supplementary Fig. 5), which is reflected by the lower binding affinity of motif IV compared with the RRKR motif (Supplementary Table 1a,b).

Taken together, the CD2CD3–IV complex reveals a unique arrangement of tandem chromodomains that are stabilized by the highly positively charged A3CT IV accommodated in their interface. A3CT IV completes the fold of CD3 by β-augmentation in a specific manner that is distinct to the CD2–RRKR_p_ interaction.

### Ligand recognition by the modified cage in CD3

The most significant difference of the CD2CD3–IV complex in comparison to classical chromodomain–ligand interactions concerns the specific ligand recognition in a ‘modified cage'. Cage modification also occurs in CD2 as seen in the cpSRP43ΔCD3–RRKR_p_ complex and is therefore a characteristic of both cpSRP43 chromodomains. In the CD2CD3–IV complex, A3CT IV adopts an extended conformation and a positively charged KRSKRKR sequence (residues 454–460) at its C terminus is threaded into CD3 (Fig. 3a and Supplementary Fig. 6). Insertion of A3CT IV as strand β1′ involves Arg458 (0 position, Fig. 3b), which is accommodated in the modified cage, and the flanking four residues Lys454–Lys457 at positions −4 to −1, and Lys459 and Arg460 at the +1 and +2 positions. Cage modification reflects the change of ligand from a methylated lysine in context of the ARKS signature sequence for classical chromodomains (Fig. 3c,d and Supplementary Fig. 5) to an arginine for CD3 and CD2. While Trp343 forms a π-cation stacking with the guanidinium group of the arginine (in an orthogonal T-shaped geometry^37^) the two other aromatic residues (tyrosines) of the classical cage are replaced by Glu318 and Asp345, which are involved in salt bridges with the caged arginine. Mutation of Trp343 to alanine drastically reduces the binding affinity (*K*_D_ of 74.6 μM) (Supplementary Table 1a). All cage residues are conserved in CD2 and CD3 (Supplementary Fig. 4a). In CD2, the cage accommodates Arg537 (0 position) of the cpSRP54 RRKR motif in an identical manner (Fig. 3b–d and Supplementary Fig. 5b). However, CD2 supplies a second cage (residues Phe267, Tyr269 and His304) for specific read-out of Arg536 (position -1) that is not conserved in CD3. Here the corresponding residues Leu317, Tyr319 and Asn356 do not form a cage for Lys457 (position −1 in A3CT IV) (Fig. 3a,d). Instead, Tyr319 is involved in stabilizing the interface with CD2 (see below).

Taken together, CD2 and CD3 interact with their charged ligands by using a modified cage and the recognition of flanking residues distinct from canonical chromodomains. While in CD2, a twinned cage reads out two neighbouring arginines, in CD3 respective residues of the second cage are involved in stabilization of the CD2–CD3 interface.

### Flanking residues determine selectivity

**Figure 4:**
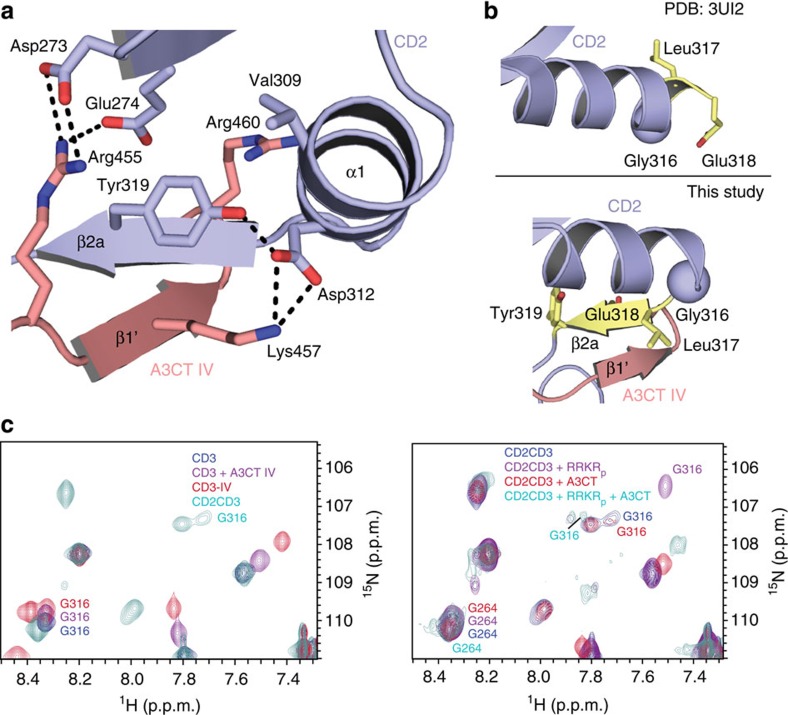
Structural basis for negative cooperativity of ligand binding to CD2 and CD3. (**a**) Close-up view on the CD2–CD3–A3CT IV interface. Tyr319 of CD3 forms the hub of the interface. Relevant salt bridges of A3CT IV to CD2 are indicated. Arg460 is fixed on the negative helix dipole of the CD2 C-terminal helix α1. (**b**) Conformational changes in the CD2–CD3 interface. Top: CD2 in the cpSRP43ΔCD3–RRKRp complex. Gly316 is integral to helix α1. Bottom: CD2CD3–IV complex structure. Gly316 serves as a hinge point (indicated as sphere) and residues 316–319 form strand β2a in the CD2–CD3 interface. (**c**) Close-up view of NMR spectra around Gly316. In the CD3 constructs (left), Gly316 is the N-terminal residue and is located at a position common for N-terminal glycines. It shifts on A3CT titration and even more if A3CT IV is connected via a GS-linker (CD3—IV). The peak position of Gly316 changes in the CD2CD3 construct (cyan), indicating a conformational change. On binding of RRKR_p_ (right, magenta) the lineshape improves and the peak shifts considerably indicating stabilization likely due to helix formation. Gly316 of RRKR_p_-preloaded CD2CD3 shifts back on addition of A3CT (cyan), according to its hinge function and the formation of strand β2a.

From the cpSRP43ΔCD3–RRKR_p_ complex it was known that CD2 recognizes the extended 531-PPGTARRKR sequence of cpSRP54 and that the residues flanking the arginine (0) and especially the arginine at the last position (+2) are important for binding^25^. Our biochemical and structural data for the CD2CD3–IV complex show that also here the extended 454-KRSKRKR sequence of Alb3 is specifically recognized. To understand how the two chromodomains discriminate between similar linear motifs from cpSRP54 and Alb3, we mutated residues from the −7 to the +2 position in A3CT IV to alanine and determined the *K*_D_ values of single and double point mutants by ITC (Supplementary Table 1a). Residues involved in salt bridges (Arg455, Lys457, Arg458) contribute the most to the binding, which is typical for an entropically unfavourable interaction and reflects the ordering of A3CT IV by formation of strand β1′ during binding. Mutation of the central Lys457 and Arg458 together decreases the dissociation constant by about ninefold (*K*_D_ of 44 μM) compared with the wild-type interaction. This highlights the importance of Arg458 (0), which is recognized within the modified cage, as the key residue in A3CT IV. Arg455 (−3) forms a salt bridge with Asp273 in CD2 (Fig. 4a) and replacement by alanine results in a sixfold reduction in binding affinity, while the mutation of Ser456 (−2) had only a minor effect (threefold reduction), probably as the small side chain of alanine still fits into place. In general, for sterical reasons chromodomains need an alanine at the −2 position of the ligand, which holds true for the cpSRP54 tail binding to CD2 and for histone tails binding to canonical chromodomains^38^ (Fig. 3b). This restriction is, however, not valid for the CD3–A3CT IV interaction due to the ‘super-twist' of strand β1′, which creates extra space for a serine residue. The importance of the −2 position for binding specificity is underlined by the recent finding that cpSRP54 tails of green algae of the chlorophyte division have a valine at this position, which specifically inhibits binding to cpSRP43 (ref. 39).

The introduction of alanine mutations at the −7 to −4 positions all show detrimental effects according to their distance to the 0 position, even though Arg451 (−7) to Ser453 (−5) are not visible in the X-ray structure. Interestingly, the corresponding region in the cpSRP54 tail (530-APP) forms a tight turn when bound to CD2, which would clash with Asp358 of CD3 (alanine in CD2) (Supplementary Fig. 5a,b). The double mutation of Lys459 and Arg460 (+1 and +2) results in a fourfold reduction of binding affinity (*K*_D_ of 20 μM). Their binding modes differ slightly in the two complexes present in the crystallographic asymmetric unit. Either the side chain of Lys459 is involved in a salt bridge with CD3 Glu352 or the guanidinium group of Arg460 is positioned on the negative dipole of the CD2 C-terminal α1 helix and stacks on the side chain of CD2 Tyr313 (Fig. 4a). These interactions highlight the importance of the preceding CD2 for the CD3–A3CT IV interaction as seen from the ITC data (Supplementary Table 1a). Intriguingly, the same binding mode utilizing the helix dipole is observed also for RRKR_p_ Arg539 at position +2 in its interaction with the Ank4 C-terminal helix^25^ (Fig. 3d). In contrast, the canonical chromodomain interaction with histone tails does not involve the +2 position, which is therefore characteristic for cpSRP43 chromodomain interactions.

Taken together, the specific interaction of CD2CD3 with A3CT IV is dominated by the flanking regions with a high-positive charge density and the formation of salt bridges. The serine in position −2 adds sterical control for A3CT IV binding. Importantly, binding of linear motifs to the cpSRP43 chromodomains involves the interface with the adjacent domain and does not solely depend on the individual chromodomains. While Ank4 contributes to binding of the cpSRP54 tail to CD2, CD2 is necessary for Alb3 binding to CD3.

### A3CT binding to CD3 shapes the CD2–CD3 interface

The biochemical and structural analysis of A3CT IV binding to CD3 showed that the interface with CD2 is an important determinant for the interaction. As A3CT IV complements the chromodomain, the ligand can be seen as part of CD3. Closer inspection shows that the interface with CD2 (330 Å^2^) is built-up by strand β2a of CD3 and by A3CT IV. Residues interacting within CD3 strand β2a include Leu317, Glu318 and Tyr319, which create a hydrophobic core within the CD2 interface surrounded by polar interactions. Tyr319 forms a hub of the interface and is triangulated by contacts with Val309 and Asp312 in helix α1, as well as with Glu274 from strand β2b (Fig. 4a). A3CT IV residues Arg455 to Lys457 complete the framing of Tyr319, with the two positively charged residues involved in salt bridges with CD2 Asp273 and Asp312, respectively. Analysis of CD2CD3 by small-angle X-ray scattering (SAXS) confirms that the domain arrangement observed between CD2 and CD3 in the crystal structure is stabilized by ligand binding (Supplementary Fig. 7 and Supplementary Table 3). These data also correlate also with previous SAXS data on cpSRP43, which indicated a reduced flexibility of CD2 with respect to the ankyrin repeats when the cpSRP54 tail was present^25^.

### Structural basis for negative cooperativity

Previous pull-down and ITC experiments showed that cpSRP43 can bind both the cpSRP54 and Alb3 tails at the same time and that the two binding events are linked with negative cooperativity^23^. Binding of A3CT to cpSRP43 loaded with RRKR_p_ leads to a five time lower affinity than to cpSRP43 alone. To analyse this in more detail, we titrated unlabelled A3CT into the pre-assembled CD2CD3–RRKR_p_ complex and followed NMR spectral changes (Supplementary Fig. 8a). CSPs observed in the domain interface on RRKR_p_ binding in CD2 helix α1 (residues 308–318) are reduced on subsequent addition of A3CT. This effect is most pronounced for Gly316, which forms a flexible hinge necessary for A3CT binding (Fig. 4b,c). Thus, the CD2–RRKR_p_ interaction is weakened as binding of A3CT to CD3 involves residues that are also affected by RRKR_p_ binding to CD2. Based on NMR secondary chemical shifts, helix α1 only extends to Asp315 in unliganded CD2CD3 (Supplementary Fig. 9). In complex with RRKRp, improvements in NMR lineshapes indicate stabilization of Gly316, which according to cpSRP43ΔCD3-RRKR_p_^25^ corresponds to extension of helix α1 by one turn (Fig. 4c). However, in complex with A3CT IV the NMR signal for Gly316 shifts back, and secondary chemical shifts (Supplementary Fig. 9) confirm formation of strand β2a as observed in CD2CD3–IV. When the NMR titration experiment is performed in opposite order and RRKR_p_ is titrated into the pre-assembled CD2CD3–IV complex (Supplementary Fig. 8b), CSPs in CD2 helix α1 are much lower than in the previous experiment. In this case, the CD3 strand β2a is already formed due to A3CT binding and residues 316 to 318 are not available for helix α1 extension.

In summary, the conformational changes in the interaction network between the tandem chromodomains provide the structural basis for negative cooperativity observed for the binding of cpSRP54 to CD2 and of Alb3 to CD3.

## Discussion

Post-translational targeting of LHCPs to the thylakoid membrane relies on the formation of the transit complex and is a specific route designed for high-throughput delivery of an abundant membrane protein. Along this route, cpSRP43 serves as the hub for multiple interactions. As central component of the transit complex it recruits cpSRP54, it serves as specific chaperone for LHCPs, and provides the docking site for the Alb3 insertase at the thylakoid membrane. The modular assembly of cpSRP43 by three chromodomains and four central ankyrin repeats is the basis for simultaneous recognition of linear motifs provided by these diverse binding partners. Previously, we deciphered the structural basis for interactions between the ankyrin repeats and the L18 peptide of the LHCPs^24^ and between CD2 and the C-terminal tail of cpSRP54 (ref. 25).

**Figure 5:**
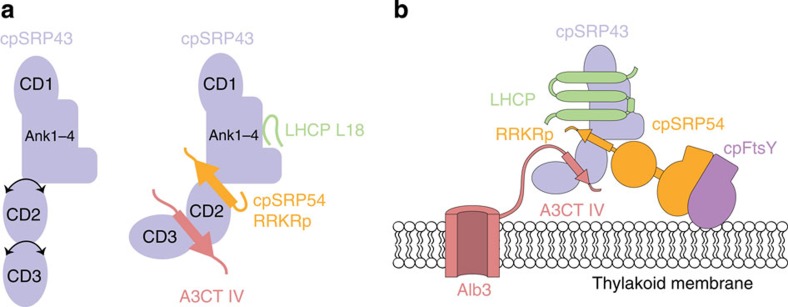
Scheme for post-translational transit complex targeting to the thylakoid membrane. (**a**) Schematic representation of cpSRP43 interactions with linear motifs in post-translational targeting. Flexibility in absence of the binding partners between ankyrin repeats and CD2, and between CD2 and CD3 is indicated with black arrows. (**b**) Scheme for transit complex interactions at the thylakoid membrane. CpSRP54 establishes the connection to the SRP receptor cpFtsY via targeting complex formation, whereas A3CT tethers the transit complex to the Alb3 insertase within the membrane.

We now complete the structural portfolio of cpSRP43 interactions by showing that cpSRP43 CD3 specifically interacts with a linear motif in the C-terminal tail of Alb3. We delineate the importance of the domain interfaces in cpSRP43 for recruitment of the transit complex to the thylakoid membrane (Fig. 5a). CD3 recognizes the positively charged motif IV with a central arginine being accommodated in a modified cage with respect to classical chromodomains. The same principle applies to the cpSRP43–cpSRP54 interaction, which is therefore the key-feature of the cpSRP43 chromodomains. Selectivity for CD2 and CD3 is achieved by adaptation of the flanking residues in the interacting linear motifs. CpSRP54 binding to CD2 involves two consecutive arginine residues in the C-terminal tail that are accommodated in a twinned cage and two consecutive proline residues that form a tight β-turn^25^. Alb3 binding to CD3 is weaker due to distortion of the β-completion and removal of the second cage. Specificity arises as the prolines of the cpSRP54 tail do not fit in CD3 and a serine in the Alb3 tail cannot be accommodated in CD2. In addition, discrimination between motifs II and IV in Alb3 is supported by the read-out of positive charges in the flanking regions of motif IV. Therefore, although the recognized linear motifs are highly similar, each of them contains unique features for distinct recognition. Likewise, preceding domains of cpSRP43 contribute to the ligand read-out in CD2 and CD3: the Ank4 repeat to cpSRP54 and CD2 to Alb3 recognition, respectively. The mode of interface arrangements is also similar and includes the negative dipole of the terminal α helices of the preceding modules that bind respective arginines of the ligands. CpSRP43 contains a third chromodomain at its N terminus (CD1), which shows high sequence and structure conservation^24^,^25^. However, CD1 has so far not been implicated in interactions with components of LHCP biogenesis. In contrast to CD2 and CD3, the modified cage of CD2 and CD3 is not present in CD1 and a preceding folded domain is missing, which apparently prevents cpSRP54 and Alb3 binding and a ligand, if any, remains to be identified. Interestingly, although A3CT IV (*K*_D_ 15 μM) contributes most to the overall affinity of A3CT (*K*_D_ 5 μM) for cpSRP43, additional low affinity interactions involving A3CT-II increase the avidity of the interaction. Previously, fluorescence complementation assays with protoplasts indicated a stronger contribution of motif II (than motif IV) to the interaction with cpSRP43 and an additional binding site in transmembrane domain five of Alb3 (TMD5)^40^. A recent study in planta described that an Alb3 truncation (including motif IV) results only in a slight reduction in LHCP accumulation compared with wild-type plants when grown under low light conditions^41^. Our structural and biochemical study focused on the interaction between cpSRP43 CD2CD3 and the Alb3 tail, but additional binding sites in full-length Alb3 may contribute to the interaction *in vivo*. The presence of multiple linear motifs in the long Alb3 tail suggests that fly-casting contributes to the interaction with cpSRP43.

Our data provide the structural basis for cpSRP43 tandem chromodomain interaction with their substrates. This interaction differs from the interactions of chromo shadow domains with their binding partners^42^. There, recognition of a central PXVXL pentapeptide requires the interface of a symmetrical chromo shadow domain dimer^42^. However, like for the cpSRP43 chromodomain interaction with cpSRP54 and Alb3, the recognition of flanking residues is also important for the interaction of chromo shadow domains with their substrates and determines specificity.

The C-terminal tails of cpSRP54 and Alb3 can bind simultaneously to cpSRP43 (ref. 23). The two binding events show negative cooperativity as binding of the first ligand lowers the affinity of the second ligand by a factor of five. Our NMR data indicate that C-terminal residues of CD2 are involved in this crosstalk and the crystal structure of CD2CD3–IV reveals Gly316 as a hinge point between the two chromodomains. When only the cpSRP54 tail is bound to CD2CD3, the helix propensity increases for residues beyond Gly316; however, when the Alb3 tail binds to CD3, residues 316–319 undergo a conformational change to a β-strand conformation and constitute the CD2–CD3 interface. Negative cooperativity might be important for membrane docking of cpSRP to Alb3 and for the handing over of LHCP to Alb3. The lower affinity observed in the Alb3 interaction might reflect the necessity of a transient membrane tether, which can be released when LHCP has been handed over to Alb3 for insertion. The negative cooperativity between the CD2 and CD3 interactions indicates inter-domain communication, which could allow for sensing the presence or absence of LHCP. However, further studies are needed to clarify the biological implications of this gain-of-function.

Alb3 belongs to the YidC/Oxa1/Alb3 family of membrane insertases^18^,^19^, which are responsible for insertion and folding of membrane proteins and their assembly into larger protein complexes^18^. YidC and Oxa1, members of the insertase family, have been implicated in co-translational membrane protein biogenesis. YidC recruits ribosomes to the plasma membrane in bacteria, while in mitochondria, which lack SRP, Oxa1-mediated ribosome docking to the inner membrane is required for efficient membrane insertion of proteins involved in oxidative phosphorylation. Oxa1 and YidC utilize their positively charged C-terminal extensions for ribosome binding^43^,^44^,^45^. The C-terminal tails of YidC in most Gram-negative bacteria are short and contain less positive charges. Fusion of an extended C-terminal tail of YidC from marine bacteria like *Rhodopirellula baltica* and *Oceanicaulis alexandrii* to *E. coli* YidC highly improved ribosome binding^21^. Both Alb3 and Oxa1 have a long C-terminal tail with pronounced clusters of positively charged residues^46^. While the details of the Oxa1–ribosome interactions are not yet resolved, Alb3 utilizes the tail to specifically interact with CD3 of the negatively charged cpSRP43. Overall, the interaction of Alb3 with cpSRP43 and Oxa1 or YidC with ribosomes appears mechanistically similar, and the evolutionary role of cpSRP43 might be to adapt the cpSRP system to post-translational targeting (Fig. 5b). The tandem array of cpSRP43 chromodomains allows interacting with both Alb3 and cpSRP54 to efficiently deliver its LHCP cargo to the membrane insertase and to regulate the targeting process by the SRP machinery.

## Methods

### Cloning

The different A3CT constructs encoding the amino acid sequence 363–462 were cloned into pET21d via NcoI/XhoI restriction sites. Single and double point mutations in A3CT were generated in pET21d using the QuikChange system (Stratagene). The cpSRP43 CD2CD3 deletion construct encoding amino acids 264–373 and the cpSRP43 CD3 deletion construct encoding amino acids 316–373 were cloned into pETtrx_1a^47^ with a cleavable tobacco etch virus (TEV) site via NcoI/XhoI restriction sites. Single point mutations in cpSRP43 were generated in the pET24a vector using the QuikChange system. The A3CT IV peptide encoding amino acid sequence 434–461 was cloned into pETgst_1a with a cleavable TEV site using NcoI and XhoI restriction sites. For the CD2CD3–IV fusion construct, A3CT IV encoding residues 440–461 was covalently linked with a (GS)_3_ to cpSRP43 CD2CD3 encoding residues 264–373. CD2CD3–IV was then cloned with a single GS-linker into pETtrx_1a via NcoI/XhoI restriction sites.

### Protein production and purification

A3CT wild-type and A3CT mutants with a C-terminal His_6_-tag were produced in *E. coli* BL21 (DE3) cells. Protein production was induced with 1 mM isopropyl-1-thio-β-D-galactopyranoside (IPTG) at an *A*_600_ of 0.8–1.0. After induction, the proteins were expressed for 16 h at 18 °C, harvested and stored at −80 °C. His-tagged A3CT pellets were resuspended in lysis buffer (100 mM Hepes/NaOH (pH 7.5), 300 mM NaCl, 5 mM MgCl_2_, 10% (v/v) glycerol, 0.02% (v/v) 1-thioglycerol). The cells were lysed with a M1–10L microfluidizer (microfluidics), the lysate was clarified and the supernatant was applied onto a 1-ml HisTrap HP column (GE Healthcare). The column was washed with washing buffer (50 mM Hepes/NaOH (pH 7.5), 300 mM NaCl, 5 mM MgCl_2_, 5% (v/v) glycerol, 0.02% (v/v) 1-thioglycerol) containing 0, 20 and 50 mM imidazole. Protein elution occurred with a buffer containing 300 mM imidazole. Protein containing fractions were pooled and subjected to a S75 26/60 size-exclusion chromatography equilibrated in 20 mM Hepes/NaOH (pH 7.5), 150 mM NaCl, 2 mM MgCl_2_ and 1 mM DTT. N-terminally His_6_-tagged cpSRP43 wild-type and cpSRP43 mutants were produced in *E. coli* Rosetta (DE3) pLysS. Cells were grown at 37 °C to a cell density of 0.6–0.8 OD per ml. Protein production was induced by 0.2 mM IPTG. After 12 h at 18 °C, the cells were harvested and stored at −80 °C. Protein production was performed as described above. N-terminally His_6_-tagged Trx–CD2CD3–IV was produced in *E. coli* Rosetta2 (DE3) pLysS cells in auto-induction medium. His_6_–Trx–CD2CD3–IV was purified as described above.

N-terminally His_6_-tagged Trx–CD2CD3 and Trx–CD3 with a cleavable TEV site were produced in *E. coli* Rosetta2 pLysS cells. Cells were grown at 37 °C to a cell density of 0.6–0.8 OD per ml. Protein production was induced by 0.2 mM IPTG. After 12 h at 18 °C, the cells were harvested and stored at −80 °C. His_6_–glutathione-S-transferase (GST)–A3CT IV peptide was produced in *E. coli* BL21 cells. Cells were grown at 37 °C and protein production was induced by 0.4 mM IPTG at *A*_600_ of 0.8–1.0. After 3 h at 37 °C, the cells were harvested and stored at −80 °C. Purification of proteins fused to Trx and GST was performed as described in a previous work^23^ with the following adaptation for A3CT IV peptide: after TEV cleavage over night, the sample was reloaded on a HisTrap column and the A3CT IV peptide was eluted in a buffer containing 50 mM Hepes/NaOH (pH 7.5), 300 mM NaCl, 5 mM MgCl_2_, 5% (v/v) glycerol, 0.02% (v/v) 1-thioglycerol and 20 mM imidazole. Protein containing fractions were pooled and the protein was loaded on a 26/60 Superdex75 column (GE Healthcare) equilibrated in gel filtration buffer (20 mM Hepes/NaOH (pH 7.5), 150 mM NaCl, 2 mM MgCl_2_).

### Protein production and purification for NMR

N-terminally His_6_-tagged Trx–CD2CD3 and Trx–CD3 were produced in *E. coli* Rosetta2 pLysS cells, and N-terminally His_6_-tagged Trx–A3CT and His_6_–GST-tagged A3CT IV peptide were produced in *E. coli* BL21 cells. The proteins were produced in M9 medium supplemented with [^15^N]H_4_Cl and ^13^C-glucose as the sole sources of nitrogen and carbon for uniformly protein labelling. After induction with 1 mM IPTG at an *A*_600_ of 0.8–1.0, the proteins were produced for 12 h at 30 °C. The proteins were purified as described previously^23^. For size-exclusion chromatography, the samples were loaded on a 26/60 Superdex75 column equilibrated in NMR buffer (20 mM Na-phosphate (pH 6.5), 150 mM NaCl).

### Isothermal titration calorimetry

ITC experiments were performed in a buffer containing 20 mM Hepes (pH 7.5), 150 mM NaCl, 5 mM MgCl_2_ and 0.25 mM TCEP (tris(2-carboxyethyl)phosphine). All protein samples and peptides were dialysed over night in this buffer and extensively degassed prior to titration. ITC experiments were conducted on a VP-ITC microcalorimeter (MicroCal, Northampton, MA) at 20 °C with 307 r.p.m. stirring. Titrations consisted of 23 injections of 12 μl aliquots of the titrant into the solution of the cell. Typical protein concentrations in the cell were 0.01–0.1 mM and 0.1–1.0 mM in the syringe. Data were analysed with Origin 7.0 software. The ITC measurements were performed in duplicate or triplicate. Peptides were purchased from PSL (Peptide Speciality Laboratories, Technology Park, Heidelberg), purified by HPLC and analysed by mass spectrometry.

### Protein crystallization and data collection

Crystals of His_6_–Trx–CD2CD3–IV were grown in an in-house automated crystallization platform at 18 °C in sitting drops containing 0.2 μl of His_6_–Trx–CD2CD3–IV (17 mg ml^−1^) and 0.2 μl of a reservoir solution consisting of 20% (w/v) PEG 3350 and 0.2 M Ca(OAc)_2_. Crystals grew as thin and fragile plates after 5 days. The crystals were cryoprotected in mother liquor containing 20% (v/v) glycerol and flash-cooled in liquid nitrogen. Data were collected at the European Synchrotron Radiation Facility (ESRF, Grenoble) on beamline ID29 at 0.992 Å and 100 K. Data were integrated and scaled with XDS^48^ and AIMLESS from the CCP4 package^49^.

Crystals of cpSRP43 CD3 were grown at 4 °C in sitting drops containing 0.2 μl of cpSRP43 CD3 complexed with A3CT IV (12 mg ml^−1^) and 0.2 μl of a reservoir solution consisting of 25% PEG 3350, 0.1 M Hepes pH 7.5 and 0.2 M MgCl_2_. Crystals containing only cpSRP43 CD3 grew as little squares after 21 days. The crystals were cryoprotected in mother liquor containing 35% PEG and 20% glycerol, flash-frozen in liquid nitrogen and measured at the ESRF on beamline ID29 at 1.033 Å and 100 K. Data were integrated and scaled with XDS^48^ and AIMLESS from the CCP4 package^49^.

### Structure determination and refinement

The construct His_6_–Trx–CD2CD3–IV crystallized in the orthorhombic space group P2_1_2_1_2 with two molecules in the asymmetric unit. The cell parameters are *a*=79.6 Å, *b*=163.7 Å, *c*=37.4 Å and the solvent content is 42%. The structure was solved by molecular replacement as implemented in PHASER^50^ using Trx (PDB code 3DXB) and one ensemble of the solution structure of cpSRP43 CD3 as a search model. The structure was refined using Phenix.refine^51^ and iterative model building in COOT^52^ and was validated with MOLPROBITY^53^ as implemented in PHENIX^51^. Ramachandran statistics for the final model show 97% of residues in most favoured regions and 3% of residues in additionally allowed regions. All structural figures were prepared with PyMOL^54^. Electrostatic surface potentials were calculated with APBS integrated in PyMOL^54^. Sequence alignments were performed with ClustalX^55^ and protein sequence conservations were performed using the ConSURF server^56^.

CpSRP43 CD3 crystallized in the cubic space group I432 with a cell axis of 103.9 Å. The solvent content was determined to 59% with one molecule in the asymmetric unit. The structure of the open CD3 was solved by molecular replacement as implemented in PHASER^50^ using the solution structure of cpSRP43 CD3 as a search model. Structure refinement was performed with Phenix.refine^51^ and iterative model building in COOT^52^. The structure was validated with MOLPROBITY^53^ as implemented in PHENIX^51^. Ramachandran statistics for the final model show 96% of residues in most favoured regions and 4% of residues in additionally allowed regions. All structural figures were prepared with PyMol^54^.

### NMR spectroscopy

Spectra were acquired at 298 K (all experiments, except backbone assignment experiments of A3CT motif IV and its titrations with CD3, which were carried out at 278 K) on Bruker Avance III NMR spectrometers equipped with cryogenic triple resonance gradient probe heads at magnetic field strengths corresponding to proton Larmor frequencies of 600 and 800 MHz. All spectra were processed with NMRPipe^57^ and analysed using Sparky^58^ and CARA (http://cara.nmr.ch). For backbone and side chain assignments HNCACB, CBCA(CO)NH and HCCH-TOCSY spectra were recorded^59^. Distance restraints were obtained from ^15^N- and ^13^C-edited 3D NOESY-HSQC spectra. Secondary structure prediction from secondary chemical shifts (Cα and Cβ) was done according to the study by Wishart and Sykes^60^. NMR CSPs of CD3, CD2CD3, A3CT and motif II/IV on titration with each other and RRKR_p_ were observed by titrating the ligand stepwise until saturation was reached. A series of ^1^H, ^15^N HSQC spectra were recorded for ^15^N-labelled CD3, CD2CD3, A3CT or motif IV with various amounts of binding partner. The following stoichiometries were used for diverse interactions (with the ^15^N-labelled partner being first): CD2CD3:A3CT (1:0.5, 1:1, 1:1.5, 1:2.7, 1:3.9); CD2CD3:RRKR (1:0.25, 1:0.35, 1:0.7, 1:1.1, 1:1.9, 1:2.8, 1:3.8); A3CT:CD2CD3 (1:0.55, 1:1.2, 1:1.8, 1:3.2); CD2CD3:A3CT:RRKR (1:3.9:0.7, 1:3.9:2.2, 1:3.9:5.2); CD2CD3:RRKR:A3CT (1:3.8:2.8); CD3:motif IV (1:0.9, 1:2.7, 1:7); motif IV:CD3 (1:1, 1:2.1, 1:3.2, 1:5.7, 1:8.4); CD3:motif II (1:0.9, 1:2.7, 1:7); CD2CD3:motif IV (1:0.9, 1:2.3, 1:7) and CD2CD3:motif II (1:0.9, 1:2.3, 1:7). All samples used in the titrations were in the same buffer to prevent CSPs based on buffer or pH effects. Affinities from NMR data could not be obtained in most cases as interactions were in the intermediate exchange regime. Interacting residues were identified by manually tracing peaks in the NMR spectra and confirmed by recording and analysing backbone assignment experiments for the bound form. Atom-specific chemical shift weighting was done according to Mulder *et al.*^61^.

The NMR structure of CD3 was calculated by automated NOE cross-peak assignment and torsion angle dynamics, using the software CYANA 3.0 (ref. 62). Automatically assigned NOEs and completeness of the NOE cross-peaks were manually inspected. Distance restraints from the CYANA calculation and TALOS+ derived^63^ were used in a water refinement calculation^64^ using ARIA 1.2 (ref. 65). Structural quality of the final ensemble of 10 structures with lowest energy was validated using the iCING web server^66^. Ramachandran statistics for the final NMR structure of CD3 show 90.9% of residues in most favoured regions and 9.1% in additionally allowed regions. The structural statistics are shown in Supplementary Table 2.

### Small-angle X-ray scattering

The constructs cpSRP43 CD2CD3 (residues 264–373) and cpSRP43 CD3–IV were produced and purified as described above. About 50 μl of CD2CD3 (5, 10, 20 mg ml^−1^) and CD2CD3 with 7 × molar excess of A3CT IV (2.5, 5, 10 mg ml^−1^), as well as buffer and buffer with the same amount of A3CT IV were measured at 298 K on a Rigaku BioSAXS1000 using a Pilatus detector. Six frames with 900-s exposure time per frame were recorded for each sample and buffer using an X-ray wavelength of *λ*=1.5418 Å. Frames showing radiation damage were removed prior to data analysis. For SAXS data collection and processing, the software SAXSLab 3.0.1r1 was used. The one-dimensional scattering intensities of samples and buffers were expressed as a function of the modulus of the scattering vector *Q=*(4*π*/*λ*)sin*θ*. Buffer intensities were subtracted from CD2CD3 samples, and buffer plus peptide intensities were subtracted from CD2CD3 plus A3CT IV samples using the software PRIMUS^67^. The radii of gyration *R*_g_ of all samples were extracted by the Guinier approximation with the same programme. *R*_g_ and *D*_max_ were also calculated from pairwise distribution functions using GNOM^68^. CRYSOL^69^ was used to fit the experimental scattering densities of CD2CD3 plus A3CT IV with the back-calculated scattering densities from the crystal structure. All statistics are summarized according to the study by Jacques *et al.*^70^ in Supplementary Table 3.

## Additional information

**Accession codes:** Protein Data Bank: coordinates and structure factors have been deposited under the accession codes 5E4W, 5E4X and 2N88. The NMR chemical shifts are deposited in the Biological Magnetic Resonance Data Bank, entries 25837 and 25838.

**How to cite this article:** Horn, A. *et al.* Structural basis for cpSRP43 chromodomain selectivity and dynamics in Alb3 insertase interaction. *Nat. Commun.* 6:8875 doi: 10.1038/ncomms9875 (2015).

## Supplementary Material

Supplementary Information Supplementary Figures 1-9, Supplementary Tables 1-3 and Supplementary References

## Figures and Tables

**Table 1:** Data collection and refinement statistics (molecular replacement).

	**Trx–CD2CD3–IV**	**CD3**
*Data collection*		
Space group	P 2_1_ 2_1_ 2	I 4 3 2
		
Cell dimensions		
*a*, *b*, *c* (Å)	79.6, 163.7, 37.4	103.9, 103.9, 103.9
*α*, *β*, *γ* (Å)	90.0, 90.0, 90.0	90.0, 90.0, 90.0
Resolution (Å)	45–2.8 (2.9–2.8)	42.4–2.75 (2.9–2.75)
*R*_pim_ (%)	11.3 (44.3)	3.7 (34.2)
*I*/*σI*	6.7 (1.9)	23.7 (3.7)
Completeness (%)	99.9 (99.8)	100.0 (100.0)
Redundancy	5.5 (5.4)	48.8 (52.2)
*Refinement*		
Resolution (Å)	45–2.8	42.4–2.75
No. of unique reflections	12,743	2,708
*R*_work_/*R*_free_ (%)	25.0/29.2	24.7/28.0
		
*No. of atoms*		
Protein	3,490	416
Ligand/ion	14	1
Water	41	16
		
*Average B-factor (Å^2^)*		
Protein	71.6	109.6
Ligand/ion	−	−
Water	−	−
		
*r.m.s. deviations*		
Bond lengths (Å)	0.003	0.004
Bond angles (°)	0.73	0.78

CD, chromodomain; Trx, thioredoxin.

Statistics for the highest resolution shell are shown in parentheses.
