# Supplementary material for: Structural basis for cpSRP43 chromodomain selectivity and dynamics in Alb3 insertase interaction
Source: Nat Commun. 2015 Nov 16;6:8875. doi: 10.1038/ncomms9875 (PMC4660199; doi:10.1038/ncomms9875)
Supplement: Supplementary Information — Supplementary Figures 1-9, Supplementary Tables 1-3 and Supplementary References [file ncomms9875-s1.pdf]

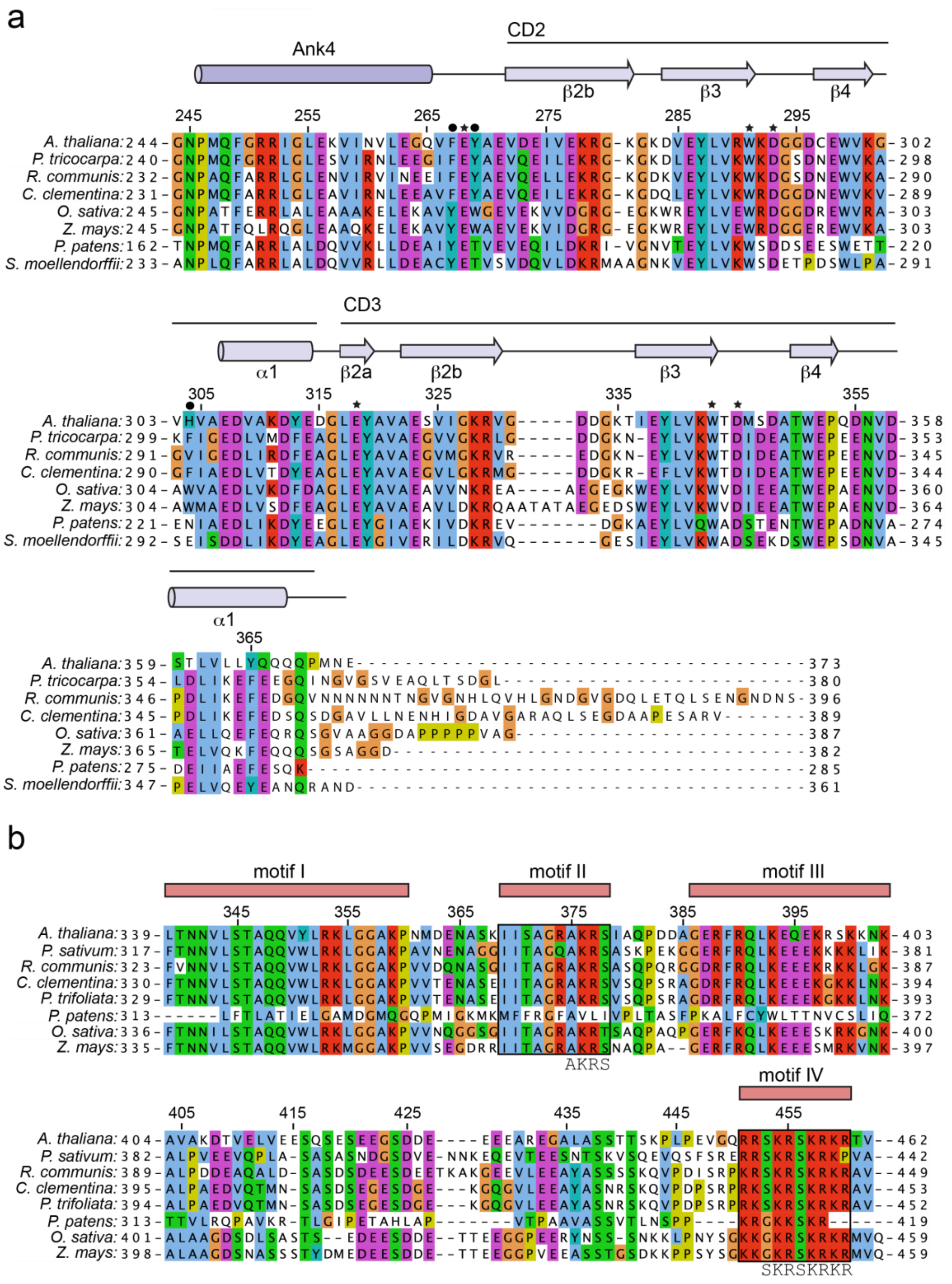

Supplementary Figure 1. Sequence alignments. (**a**) Sequence alignment of cpSRP43 CD2CD3 from representative organisms. Secondary structure elements based on the crystal structure of cpSRP43 CD2CD3 are depicted on top. Cage residues are marked with a black asterisk and residues of the second cage within CD2 are marked with a black dot. (**b**) Sequence alignment of A3CT. The four conserved sequence motifs I to IV are shown in salmon. The ARKS-like signature sequences within motif II and IV (boxed) are indicated.

a

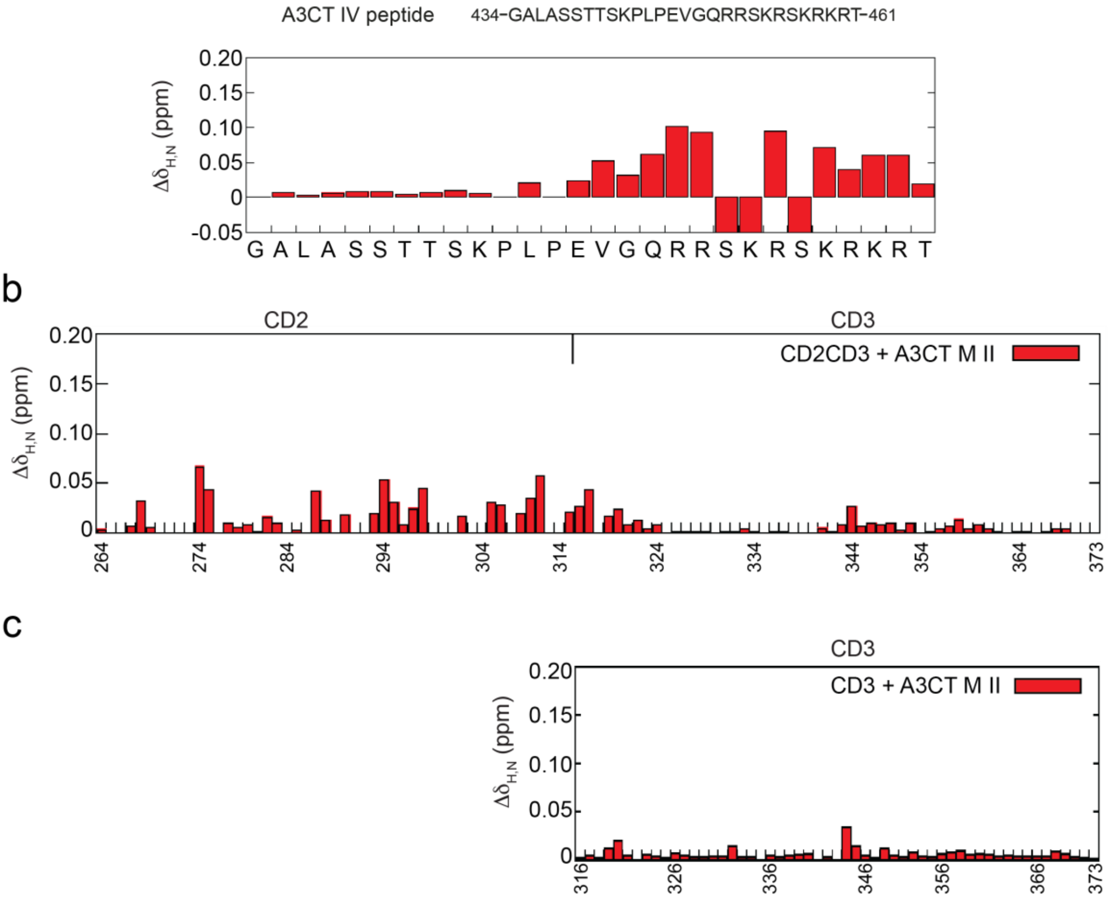

Supplementary Figure 2. NMR titrations with A3CT motifs. (**a**) CSPs for the A3CT IV peptide upon titration with CD3 showing major shifts for motif IV and preceding residues. CSPs for residues that are affected but where the bound state could not be assigned were set to a negative value of -0.05. (**b**) and (**c**) CSPs for CD2CD3 and CD3 upon titration with A3CT motif II. No significant CSPs are observed, indicating no binding of motif II.

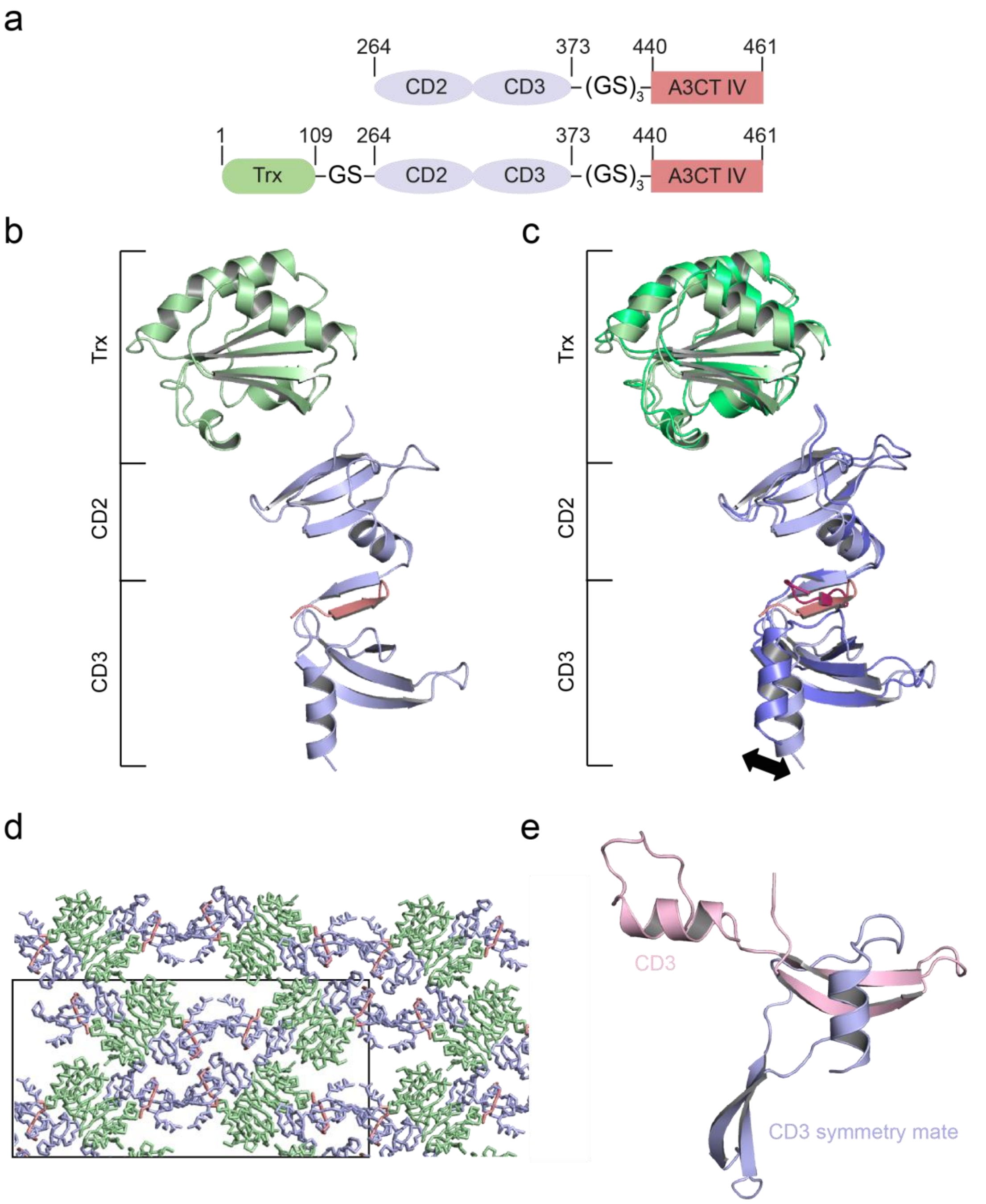

Supplementary Figure 3. Crystal structure of the Trx-CD2CD3-IV complex. (**a**) Schematic representation of the fusion constructs used for crystallization. (**b**) Crystal structure of the Trx-CD2CD3-IV complex. Trx is shown in light green, CD2CD3 in light blue and A3CT IV in salmon. (**c**) Superposition of the two molecules within the asymmetric unit of the Trx-CD2CD3-IV complex. In molecule 1 cpSRP43 CD3 is slightly tilted compared to cpSRP43 CD3 in molecule 2 (rotation is indicated by an arrow). (**d**) Crystal packing in the Trx-CD2CD3-IV complex showing that most crystal contacts are formed by the Trx-carrier. The unit cell is depicted. (**e**) Crystal structure of unliganded cpSRP43 CD3 revealing a helix swapping between two CD3 molecules.

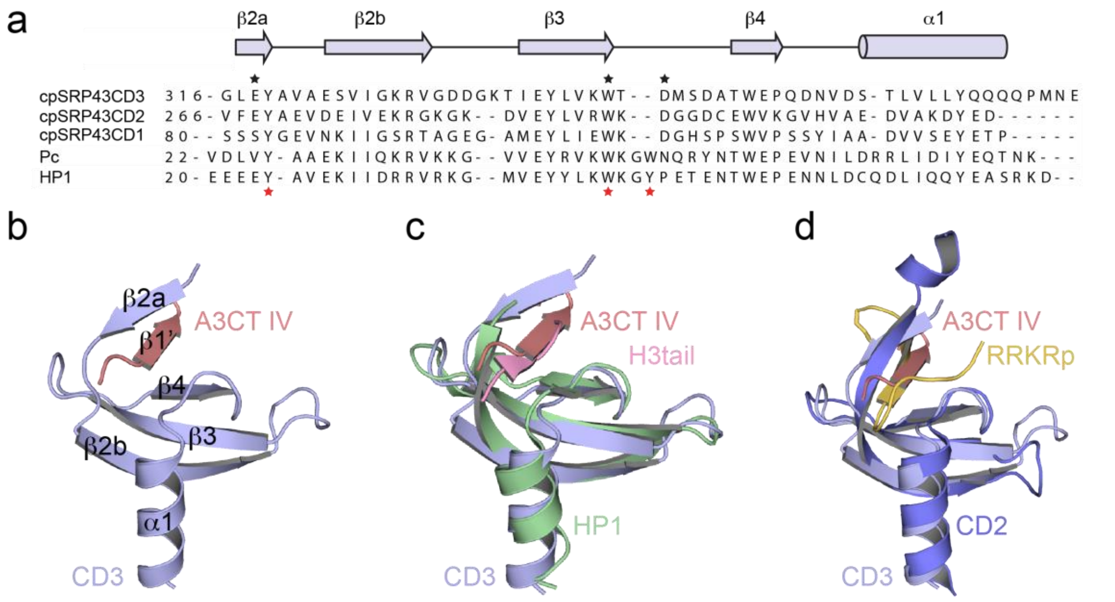

Supplementary Figure 4. Comparison of chromodomain-ligand complexes. (**a**) Structure-based sequence alignment of cpSRP43 chromodomains (CD1 to CD3) and the chromodomains of Polycomb (Pc) and Heterochromatin Protein 1 (HP1). Secondary structure elements are depicted in blue for CD3. Aromatic cage residues in canonical chromodomains are marked with a red asterisk. Corresponding residues in cpSRP43 chromodomains are marked with a black asterisk (modified cages). (**b**) Structure of cpSRP43 CD3 in complex with A3CT IV as part of the Trx-CD2CD3-IV complex. (**c**) Superposition of cpSRP43 CD3 bound to A3CT IV with HP1 in complex with histone tail H3K9me3 (pink) (green, PDB: 1KNE[1]). (**d**) Superposition of cpSRP43 CD3 bound to A3CT IV with CD2 (dark blue) in complex with RRKRp (yellow, PDB: 3UI2[2]).

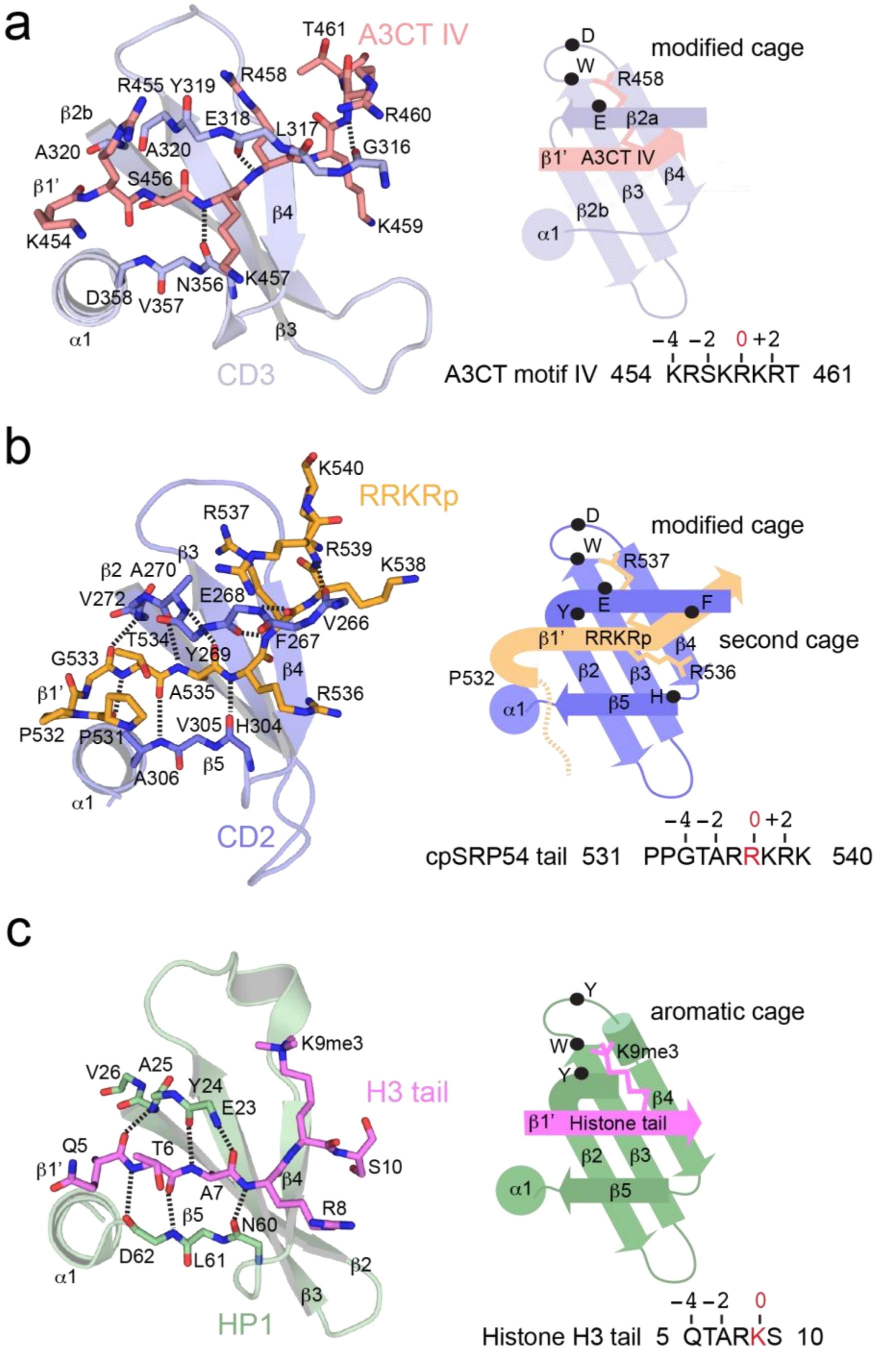

Supplementary Figure 5. Substrate binding to chromodomains. (**a**) Close-up view of the backbone interactions of CD3 with A3CT IV (left). Hydrogen bonds (in total 3) are indicated with black dashed lines. Scheme for β-completion of CD3 by A3CT IV (right). Only one cage (marked by dots) is formed accommodating arginine 458 in the 0 position and strand β5 is not present. (**b**) Analogous presentation for CD2 interactions with RRKRp (left). RRKRp binding to CD2 involves eight main chain hydrogen bonds. The second cage recognizes the arginine in the -1 position. (**c**) Close-up view and scheme for HP1 in complex with H3K9me3 (left). Residues beyond the +1 position are not read-out.

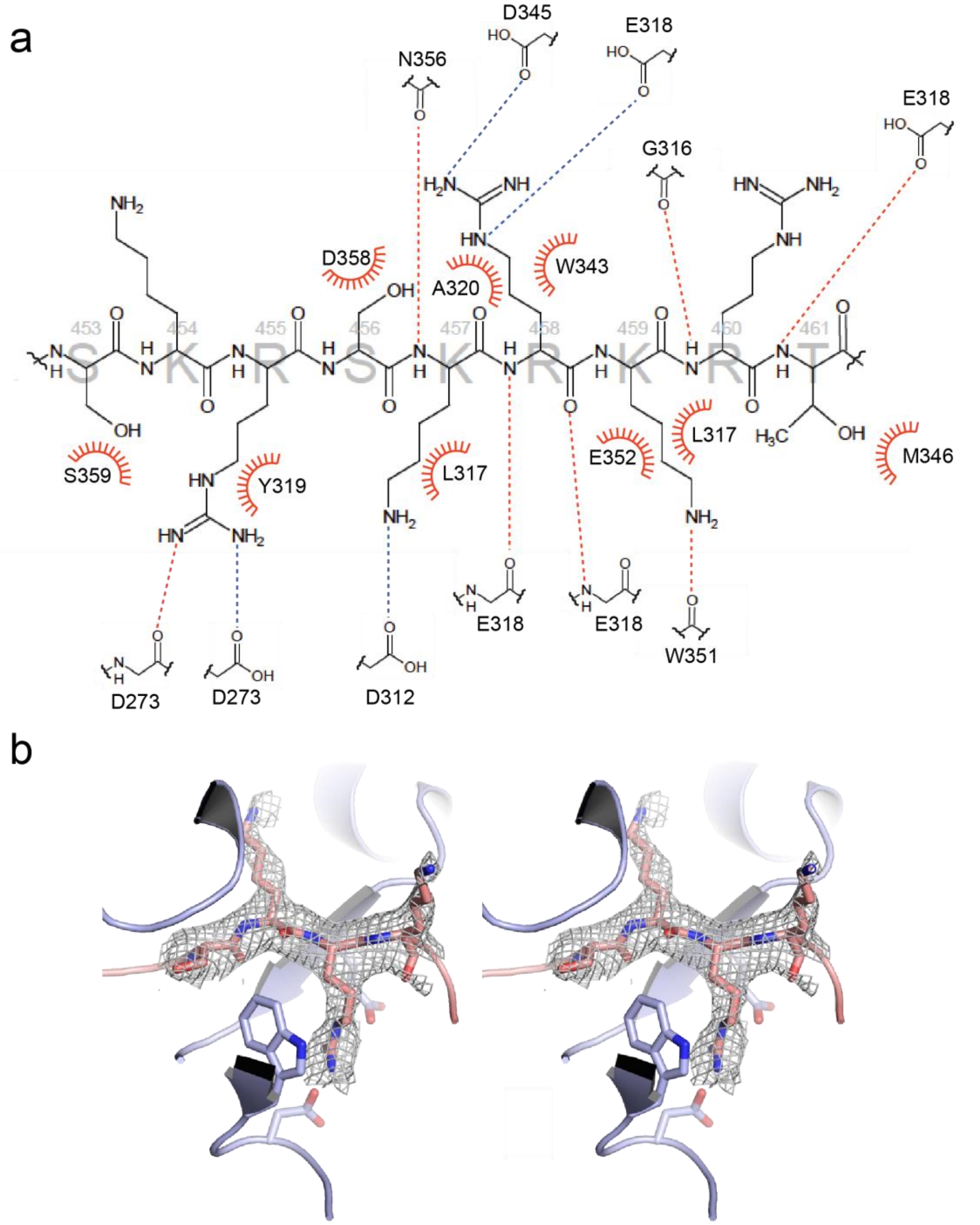

Supplementary Figure 6. Schematic representation of all interactions between cpSRP43 CD2CD3 and A3CT IV. (**a**) Hydrogen bonds to the backbone are depicted with red dashed lines and to side chains with blue dashed lines. Van-der-Waal interactions are shown as red half-circles. (**b**) Stereoview of A3CT IV bound to the CD2-CD3 interface. CD2CD3 (light blue) is shown in cartoon representation, A3CT IV (salmon) is shown in stick representation. The final 2mFo-DFc electron density map contoured at 1.0 σ is shown for the key residues in A3CT IV.

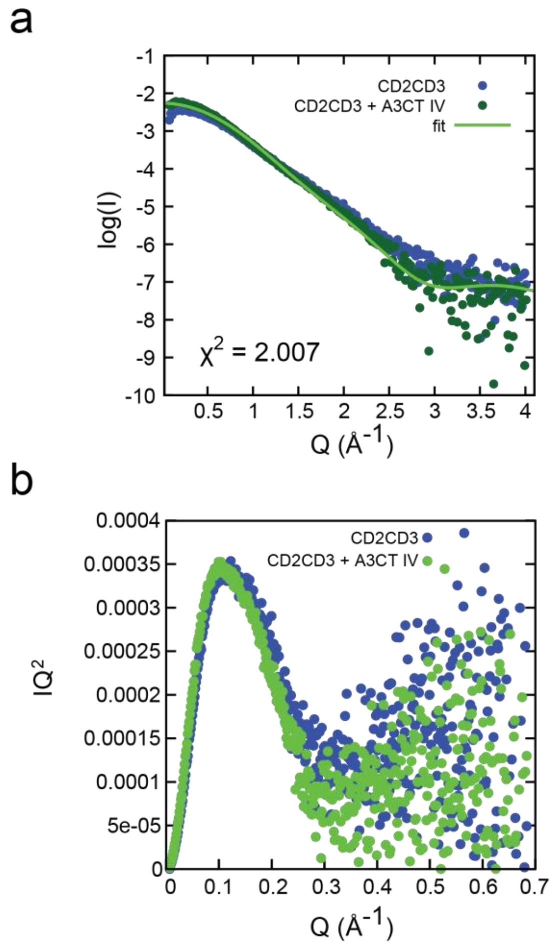

Supplementary Figure 7. Small-angle X-ray scattering (SAXS) data. (**a**) SAXS of CD2CD3 (blue dots) and bound to A3CT IV (green dots). The light green line indicates the back-calculated scattering densities from the crystal structure of CD2CD3 bound to A3CT IV and fits the experimental scattering densities well with a $\chi^2 = 2.007$. (**b**) Kratky plot representation of the scattering data from a) indicates that CD2CD3 bound to A3CT IV (green) is less flexible as the curve goes back to the baseline a bit further than the curve for CD2CD3 (blue).

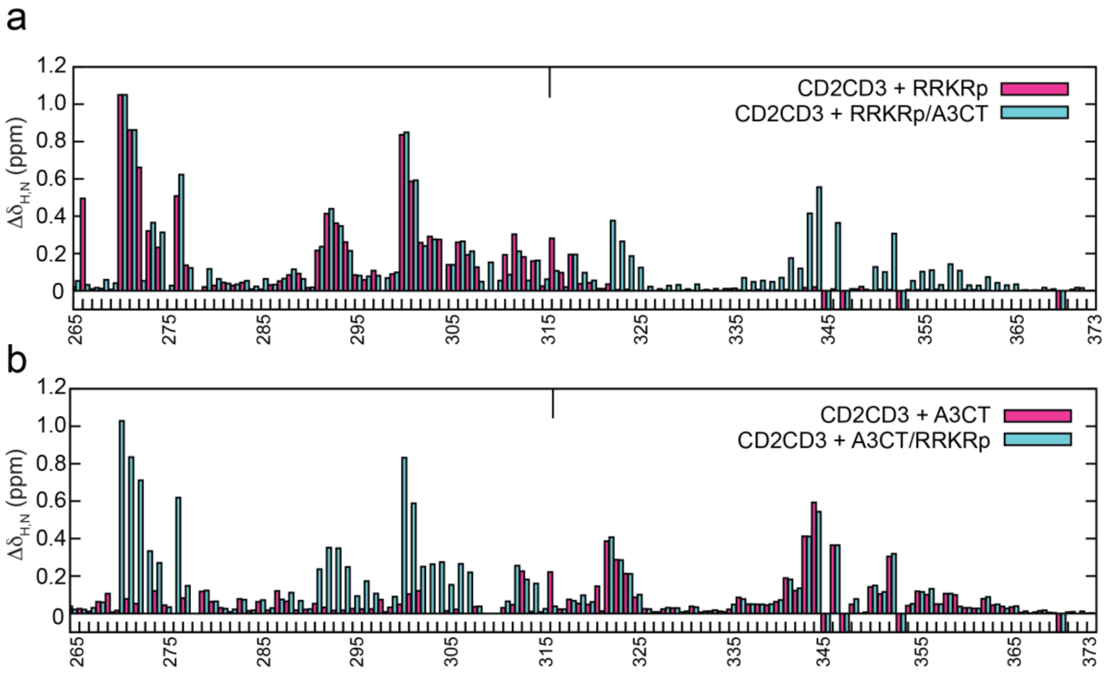

Supplementary Figure 8. Analysis of negative cooperativity in chromo-domain-ligand interaction by NMR. (**a**) CSPs for CD2CD3 upon subsequent titration with RRKRp (magenta) and A3CT (turquoise) and (**b**) CSPs for CD2CD3 upon reverse titration with A3CT (magenta) and RRKRp (turquoise). Major shifts occur for RRKRp binding to CD2 and for A3CT binding to CD3. Decreasing signals upon titration of the second ligand are visible in the connecting region between CD2 and CD3 most pronounced for residue 316. Chemical shifts for residues that are affected but where the bound state could not be assigned are set to a negative value of -0.1.

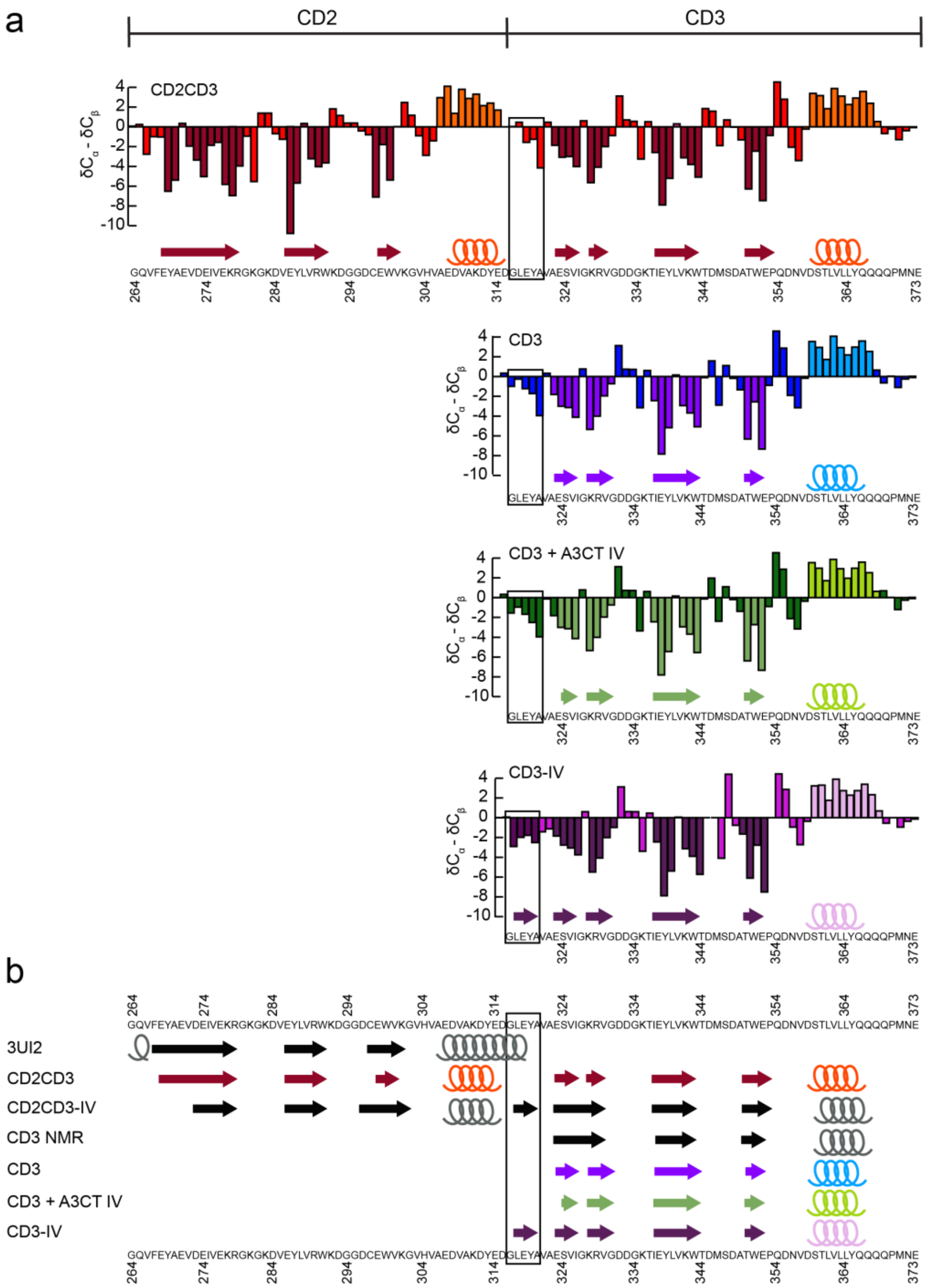

Supplementary Figure 9: Secondary chemical shifts. (**a**) Secondary structure predictions for CD2CD3 (red), CD3 (blue), CD3 with A3CT IV (green), and CD3 fused to the A3CT IV via a GS-linker (magenta) based on ^{13}C chemical shifts (C_α and C_β). The β-strand propensity (negative values) of strand β2 (boxed, residues 316-320) increases from the apo state, via A3CT IV bound, to A3CT IV fused. (**b**) Comparison of structure based secondary structures from cpSRP43ΔCD3-RRKR$_p$ (3UI2 (ref.[2])), CD2CD3-IV and CD3, as well as from predictions based on secondary chemical shifts according a). Residues 316-320 undergo a conformational change from α-helix to β-strand formation upon A3CT IV binding.

Supplementary Table 1a

Analysis of the cpSRP43-A3CT interaction by Isothermal Titration Calorimetry (ITC)

cell	syringe	K_D in µM	n	rel. aff.	ΔH kcal mol^{-1}	TΔS kcal mol^{-1}	ΔG kcal mol^{-1}
A3CT II-IV	cpSRP43	5.1 (±0.2)	1.05 (±0.01)	1.00	-21.3 (±0.1)	-14.2	-7.1
A3CT II-IV	cpSRP43 CD2CD3	20.6 (±1.3)	0.97 (±0.01)	0.25	-16.7 (±0.3)	-10.4	-6.3
A3CT IIp	cpSRP43	150.0 (±46.5)	1.09 (±0.03)	0.03	-20.3 (±2.8)	-15.2	-5.1
A3CT IVp	cpSRP43	14.8 (±0.5)	1.01 (±0.01)	0.34	-23.3 (±0.2)	-16.9	-6.4
A3CT II-IV R451A R452A	cpSRP43	15.9 (±0.2)	1.02 (±0.01)	0.32	-24.9 (±0.1)	-18.5	-6.4
A3CT II-IV S453A	cpSRP43	17.2 (±0.4)	1.07 (±0.01)	0.30	-20.0 (±0.1)	-13.5	-6.5
A3CT II-IV K454A	cpSRP43	18.4 (±1.6)	0.97 (±0.01)	0.28	-19.9 (±0.5)	-13.5	-6.4
A3CT II-IV R455A	cpSRP43	30.1 (±2.1)	0.95 (±0.02)	0.17	-21.3 (±0.5)	-15.2	-6.1
A3CT II-IV S456A	cpSRP43	15.3 (±0.5)	1.08 (±0.01)	0.33	-18.3 (±0.1)	-11.8	-6.5
A3CT II-IV K457A R458A	cpSRP43	44.2 (±0.7)	1.07 (±0.01)	0.11	-24.2 (±0.3)	-18.4	-5.8
A3CT II-IV K459A R460A	cpSRP43	20.7 (±0.3)	1.02 (±0.01)	0.25	-24.8 (±0.1)	-18.6	-6.2
A3CT II-IV	cpSRP43 W343A	74.6 (±18.8)	1.04 (±0.23)	0.07	-56.1 (±18.3)	-50.4	-5.7
A3CT IVp	cpSRP43 CD2CD3	23.1 (±3.0)	1.09 (±0.02)	0.22	-11.2 (±0.2)	-5.0	-6.2
A3CT IVp	cpSRP43 CD3	52.8 (±22.4)	1.28 (±0.08)	0.10	-6.7 (±0.2)	-0.4	-6.3

Supplementary Table 1b

Analysis of the cpSRP43-cpSRP54 interaction by ITC

cell	syringe	K_D in µM	n	ΔH kcal mol^{-1}	TΔS kcal mol^{-1}	ΔG kcal mol^{-1}
cpSRP43Δ3	cpSRP54 RRKRp	0.4* (±0.1)	0.88 (±0.01)	-23.1 (±0.3)	-14.4	-8.7
cpSRP43 CD2	cpSRP54 RRKRp	6.4* (±0.2)	0.96 (±0.00)	-26.7 (±0.1)	-19.6	-7.1
cpSRP43 CD2CD3	cpSRP54 RRKRp	8.6 (±1.9)	1.08 (±0.04)	-34.7 (±1.7)	-27.9	-6.8
cpSRP43 CD3	cpSRP54 RRKRp		no binding observed			

* K_Ds published previously in Holdermann *et al.*, 2012[2]

Supplementary Table 2

NMR and refinement statistics for CD3

**NMR distance & dihedral constraints**	
Distance restraints	
Total NOE	792
Intra-residue	219
Inter-residue	573
Sequential ($\lvert i-j \rvert = 1$)	213
Medium-range ($\lvert i-j \rvert < 4$)	109
Long-range ($\lvert i-j \rvert > 5$)	251
Intermolecular	792
Hydrogen bonds	12
Total dihedral angle restraints	85
phi	42
psi	43
**Structure statistics**	
Violations (mean and s.d.)	
Distance contraints (Å)	0.009 ± 0.002
Dihedral angle constraints (°)	0.403 ± 0.094
Max. dihedral angle violation (°)	1.22
Max. distance constraint violation (Å)	0.07
Deviations from idealized geometry	
Bond lengths (Å)	0.002 ± 0.0005
Bond angles (°)	0.4 ± 0.09
Impropers (°)	0.4 ± 0.17
Average pairwise r.m.s.d.** (Å)	
Heavy atom (Å)	0.73 ± 0.08
Backbone (Å)	0.32 ± 0.07

**Pairwise r.m.s.d. was calculated among 10 refined structures

Supplementary Table 3

Small angle X-ray scattering (SAXS) statistics

	**CD2CD3 free**	**CD2CD3 + motif IV**
Data collection		
Instrument	Rigaku, BioSAXS1000	Rigaku, BioSAXS1000
Beam geometry	Point focus	Point focus
Wavelength (Å)	1.5418	1.5418
Q range ($Å^{-1}$)	0.0066-0.45	0.0066-0.45
Exposure time (s)	5400	5400
Concentration range (mg ml^{-1})	5-20	2.5-10
Temperature (K)	298	298
Structural parameters		
I(0) (cm^{-1}) from P(r)	0.1 ± 0.001	0.11 ± 0.001
R_g (Å) from P(r)	18.5 ± 0.003	18.5 ± 0.005
I(0) (cm^{-1}) from Guinier	0.093 ± 0.001	0.114 ± 0.001
R_g (Å) from Guinier	17.6 ± 0.215	19.6 ± 0.114
D_{max} (Å)	61.7	64.5
Porod volume estimate ($Å^3$)	17666	21166
Software employed		
Primary data reduction	SAXSLab 3.0.1r1	SAXSLab 3.0.1r1
Data processing	PRIMUS	PRIMUS
Computation of model intensities	CRYSOL	CRYSOL

20 models have been calculated with DAMMIF[6,7]. An average DAMMIF model has been used as seed for DAMMIN, where additional 20 models have been calculated with a spatial discrepancy and an averaged normalized standard deviation of 0.588 ± 0.024, of which two has been discarded for CD2CD3 free, and 0.534 ± 0.021, of which one model has been discarded.

Supplementary References

1 Jacobs, S. A. & Khorasanizadeh, S. Structure of HP1 chromodomain bound to a lysine 9-methylated histone H3 tail. *Science* **295**, 2080-2083 (2002).

2 Holdermann, I. *et al.* Chromodomains read the arginine code of post-translational targeting. *Nature Structural & Molecular Biology* **19**, 260-263 (2012).

3 Shen, Y., Delaglio, F., Cornilescu, G. & Bax, A. TALOS+: a hybrid method for predicting protein backbone torsion angles from NMR chemical shifts. *Journal of biomolecular NMR* **44**, 213-223 (2009).

4 Laskowski, R. A., MacArthur, M. W., Moss, D. S. & Thornton, J. M. PROCHECK - a program to check for stereochemical quality of protein structures. *J. Appl. Cryst.* **26**, 283-291 (1993).

5 Hooft, R. W., Vriend, G., Sander, C. & Abola, E. E. Errors in protein structures. *Nature* **381**, 272 (1996).

6 Franke, D. & Svergun, D. I. DAMMIF, a program for rapid ab-initio shape determination in small-angle scattering *J. Appl. Cryst.* **42**, 342-346 (2009).

7 Svergun, D. I. Restoring low resolution structure of biological mcromolecules from solution scattering using simulated annealing. *Biophys. J.*, 2879-2886 (1999).
